# Carrageenan-Based Crowding and Confinement Combination Approach to Increase Collagen Deposition for In Vitro Tissue Development

**DOI:** 10.3390/gels9090705

**Published:** 2023-09-01

**Authors:** Joseph Krebs, Samuel Stealey, Alyssa Brown, Austin Krohn, Silviya Petrova Zustiak, Natasha Case

**Affiliations:** 1 Department of Biomedical Engineering, Saint Louis University, Saint Louis, MO 63103, USAsilviya.zustiak@slu.edu (S.P.Z.); 2 Department of Physiology and Pharmacology, School of Medicine, Saint Louis University, Saint Louis, MO 63104, USA

**Keywords:** macromolecular crowding, hydrogel, collagen, extracellular matrix synthesis

## Abstract

Connective tissue models grown from cell monolayers can be instrumental in a variety of biomedical fields such as drug screening, wound healing, and regenerative engineering. However, while connective tissues contain abundant fibrillar collagen, achieving a sufficient assembly and retention of fibrillar collagen in vitro is challenging. Unlike the dilute cell culture environment, the body’s environment is characterized by a high density of soluble macromolecules (crowding) and macromolecular networks (confinement), which contribute to extracellular matrix (ECM) assembly in vivo. Consequently, macromolecular crowding (MMC) has been successfully used to enhance the processing of type I procollagen, leading to significant increases in fibrillar collagen assembly and accumulation during in vitro culture of a variety of cell types. In this study, we developed a combination approach using a carrageenan hydrogel, which released soluble macromolecules and served as a confinement barrier. We first evaluated the local carrageenan release and then confirmed the effectiveness of this combination approach on collagen accumulation by the human MG-63 bone cell line. Additionally, computational modeling of oxygen and glucose transport within the culture system showed no negative effects of the hydrogel and its releasates on cell viability.

## 1. Introduction

There is a growing need for engineered thin connective tissues that are structurally and compositionally similar to the native tissue of interest. Thin tissues grown from cell monolayers without the use of a scaffold can be used as in vitro models for screening of pharmacologic agents and for evaluating the differentiation potential of various types of stem cells [1]. Additionally, thin tissues have been developed for the treatment of injured skin, subcutaneous connective tissue, adipose tissue, and periosteum [2]. A key consideration for the usefulness of a thin tissue model is that the de novo tissue grown in the in vitro environment replicates the native tissue composition and structure. In particular, a common characteristic for connective tissues in the body is that they contain fibrillar collagen. However, achieving an abundant, collagen-rich extracellular matrix (ECM) during in vitro culture is challenging, due in part to insufficient assembly and retention of fibrillar collagen [3].

In comparing the physiological extracellular space where tissues grow in the body to the in vitro culture environment, one critical difference is the lack of biological macromolecules within the in vitro environment. Macromolecules are a ubiquitous component of all fluid compartments within the body, with some macromolecules moving freely in solution and others present within an interconnected, insoluble, hydrophilic network permeated by the aqueous solvent [4]. These macromolecules, through repulsive interactions, reduce the volume of solvent within which transport, interactions, and reactions of solutes can occur [5]. The effects of macromolecules on solute transport and reactions in fluid medium through excluded volume effects are generally described by the biophysical concepts of macromolecular crowding and confinement. Crowding applies to fluid environments where macromolecules are present at a high total volume fraction and freely move in solution, while confinement applies to fluid environments in which structural obstacles to solute transport exist [6].

The in vivo environment within tissues is both a crowded and a confined environment. Developing approaches to apply these principles during in vitro culture could be beneficial for tissue development. Previous studies have demonstrated that macromolecular crowding (MMC) of the culture medium through the addition of various types of soluble macromolecules enhances the processing of type I procollagen, leading to significant increases in fibrillar collagen assembly and accumulation during in vitro culture of a variety of cell types [7,8]. A confinement approach has been evaluated previously by one group, with experiments using an agarose hydrogel overlay showing an improvement for in vitro collagen deposition [9].

Using a hydrogel as a confinement barrier would provide a simple, easy, and repeatable method for creation of a physical barrier. Effectiveness of this approach in supporting collagen deposition and cell viability would depend upon the ability of the hydrogel to function as a selectively permeable barrier that would allow exchange of nutrients and metabolites while limiting transport of procollagen molecules. Polysaccharides sourced from marine organisms, including agarose, alginate, chitosan, and two types of carrageenan (CR), have been used to create hydrogels and various types of scaffolds for biomedical applications [10].

The CR family consists of linear sulfated polysaccharides derived from red algae. There are multiple isoforms based on the number and position of ester sulfate groups and content of 3,6-anhydro-galactose [11]. While commonly used in the food industry as a thickening and stabilizing agent, CR has more recently been utilized in a variety of drug delivery applications [12]. The κ-isoform of CR has also been used to form hydrogels for cell encapsulation and growth factor delivery [13,14]. CR was chosen as the hydrogel material because it is non-toxic, hydrophilic, and biodegradable, and has been used extensively as an MMC reagent including in cultures with fibroblasts, osteoblasts, and tenocytes [15,16]. Compared to other common crowding agents, CR has been shown to induce the highest collagen type I deposition due to its negative charge and inherent polydispersity [16]. CR hydrogels may degrade via enzymatic hydrolysis and/or acid-based hydrolysis [17], thereby releasing soluble polymer in the cell culture medium and contributing to crowding in addition to confinement. 

Given the previously demonstrated improvement in collagen deposition when applying a MMC or confinement approach individually, we hypothesized that including these approaches together within a culture system would combine the benefits of both and lead to similar or higher collagen deposition than MMC alone. In this study, we developed a combination MMC and confinement approach using a degradable carrageenan (CR) hydrogel to both offer a confinement barrier and be a source of MMC reagents through degradation. We compared two types of CR hydrogel formulations: a pure κ-CR gel and a κ/λ-CR gel prepared from a commercially available CR powder described as a mixture of predominantly κ-CR with lesser amounts of λ-CR. Our first study objective was to characterize the degradation of the CR hydrogels in the culture system to determine local CR release. Since our long-term focus for using this combination approach was the development of a periosteal tissue model, our second objective was to evaluate the effectiveness of this combination approach to increase collagen accumulation by the human MG-63 bone cell line. The MG-63 cell line, which originates from an osteosarcoma, has been used as an osteoblast-like cell model [18] and produces collagen and osteogenic proteins in vitro [19,20]. This cell line has been used in prior studies to test effects of various factors, including mechanical stimuli and responses to biomaterials [21,22,23]. To be a useful approach, the confinement culture system should sustain appropriate nutrient concentrations to support cell viability and procollagen secretion. The final objective of the study was to develop models of glucose and oxygen transport within the confinement culture system to quantify spatial variations in nutrient concentrations, and, in the case of glucose, to also describe time-dependent variations.

## 2. Results

### 2.1. Hydrogel Degradation

**Figure 1:**
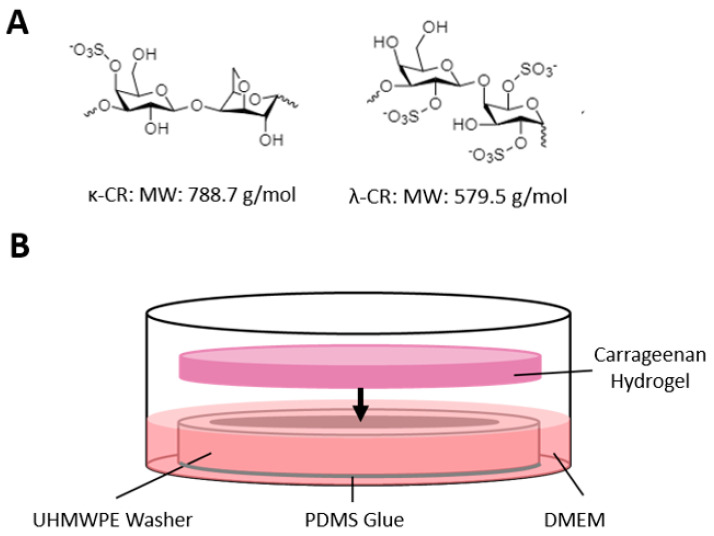
Schematic of Experimental Set-Up. (**A**) Chemical structures of λ-CR and κ-CR respectively. (**B**) Schematic of cellular confinement experiments, where the culture medium was confined radially by an UHMWPE washer and vertically by a CR hydrogel.

A combination MMC and confinement approach was achieved by overlaying CR hydrogel over 2D cell culture radially confined by an ultra-high-molecular-weight polyethylene (UHMWPE) washer (Figure 1). Two CR hydrogel formulations were investigated for their utility in achieving an MMC effect: κ-CR and κ/λ-CR.

**Figure 2:**
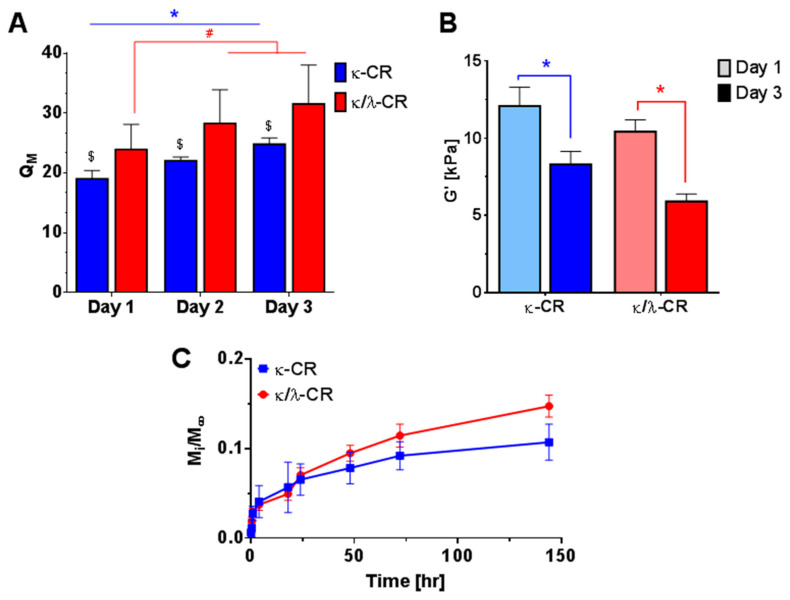
Characterization of Hydrogel Degradation. (**A**) Swelling ratio of κ-CR and κ/λ -CR hydrogels following 1, 2, or 3 days of incubation in PBS. # indicates statistically significant difference between both Day 2 and Day 3 from the Day 1 group for κ/λ-CR, while * indicates statistically significant difference between all groups of κ-CR, and $ indicates significant difference between groups of κ-CR and κ/λ-CR on each respective day (*n* = 4, *p* < 0.05) (**B**) Storage modulus at angular frequency of 5 rad/s for κ-CR and κ/λ-CR hydrogels following 1 or 3 days of incubation in PBS. * indicates statistically significant difference between Day 1 and Day 3 of κ-CR or κ/λ-CR (*n* = 4, *p* < 0.05). (**C**) Fractional release profiles of κ-CR and κ/λ-CR hydrogels measured by absorbance at 500 nm and Equation (1), where *M_i_* is the mass released at time *i* and *M_ꚙ_* is the total CR mass in the hydrogel.

κ-CR and κ/λ-CR hydrogel degradation determined the degree of CR released to produce the desired MMC effect in the culture medium above the cell layer, and hydrogel degradation was characterized using multiple measurements (Figure 2). First, we measured the swelling ratio, *Q_M_*, where gel swelling is expected to increase with time due to bulk degradation. As expected, *Q_M_* increased similarly for both κ-CR and κ/λ-CR gels from Day 1 to Day 3 by 23% and 24%, respectively (Figure 2A). However, while the change in swelling was the same for both gels, suggesting similar degradation kinetics, κ/λ-CR hydrogels showed a larger *Q_M_* than κ-CR hydrogels for each day tested, suggesting a higher crosslink density for the κ-CR hydrogels. Next, the change in hydrogel mechanical properties was used as an indirect measurement of hydrogel degradation, where degradation was expected to lead to gel softening (Figure 2B). As expected, the hydrogel storage modulus, *G*′, decreased for both κ-CR and κ/λ-CR hydrogels between Day 1 and Day 3 (31% and 43% decrease for κ-CR and κ/λ-CR, respectively). For both days tested, κ-CR hydrogels were stiffer than κ/λ-CR hydrogels, again suggesting a higher crosslink density for these hydrogels. It is of note that hydrogel mechanical properties and swelling are inversely related [24]. It was also shown that the UV-sterilization process did not affect CR hydrogel stiffness (Supplemental Figure S1).

Lastly, a direct measurement of hydrogel degradation was performed by following polymer release from the hydrogels using UV-vis spectroscopy (Figure 2C). Specifically, release of polymer molecules with hydrogel degradation led to accumulation of CR in the surrounding PBS, which could be measured by increased optical absorbance in PBS samples and quantified with prepared CR standards. The release experiments indicated a faster degradation for κ/λ-CR hydrogels compared to κ-CR hydrogels. Specifically, κ/λ-CR hydrogels showed a 9% and a 26% release at 2 and 6 days of incubation, respectively, while released mass for κ-CR hydrogels was 7% and 15% at 2 and 6 days, respectively. An initial burst release was observed for both κ-CR and κ/λ-CR hydrogels, which can be attributed to the steep initial concentration gradient between the polymer and the surrounding medium, leading to quickened release of polymer molecules. The burst release could also be attributed to polymer molecules not fully incorporated into the hydrogel network and, hence, able to quickly diffuse out or be washed off the hydrogel surface upon incubation in PBS [25]. Here, CR hydrogel degradation could likely be attributed to acid hydrolysis, as these degradation studies were performed in the absence of enzymes [26].

### 2.2. Fluorescence Correlation Spectroscopy (FCS)

**Figure 3:**
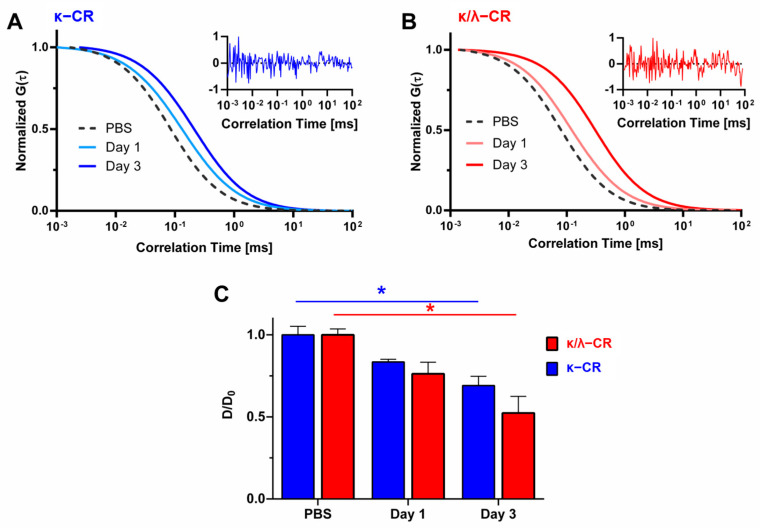
Measurements of Hindered Diffusion by Hydrogel Degradation. Representative normalized autocorrelation functions, *G*(*τ*), of labeled ribonuclease A in κ/λ-CR (**A**) or κ-CR (**B**) following 1 or 3 days of incubation in PBS. Insets represent typical residuals for Day 3 measurements. (**C**) Normalized diffusion coefficients of labeled ribonuclease A in κ/λ-CR or κ-CR hydrogels following 1 or 3 days of incubation in PBS. Diffusion coefficients (*D*) were normalized to ribonuclease A in PBS (*D*_0_). * indicates statistically significant difference between all three conditions for either κ/λ-CR or κ-CR (*n* = 4, *p* < 0.05).

FCS was used to assess potential effects of CR release with hydrogel degradation on diffusivity of solutes within the surrounding buffer (Figure 3). FCS analysis was performed on labelled RNase A as a model protein in hydrogel-conditioned PBS following 1 and 3 days of CR hydrogel incubation and subsequent CR polymer release. The rightward shift of the normalized autocorrelation functions observed when using the gel-conditioned buffer indicated hindered diffusion of RNase A caused by release of CR molecules into solution (Figure 3A for κ-CR, Figure 3B for κ/λ-CR). The diffusivities for RNase A in the hydrogel releasates were quantified and normalized by RNase diffusivity in PBS (Figure 3C), with significantly decreased diffusion of RNase A occurring for each type of releasate compared to PBS alone. As expected, the reduction in diffusivity was influenced by hydrogel incubation time as more CR polymer was released from each hydrogel. κ/λ-CR gel-conditioned buffer showed a 1.45-fold decrease in RNase A diffusivity from Day 1 to Day 3, while κ-CR conditioned buffer was reduced 1.21-fold. Following 3 days of incubation, RNase A diffusivity in κ/λ-CR releasates was significantly lower compared to κ-CR releasates.

### 2.3. CR Gel Confinement Effects on 2D Cell Cultures

**Figure 4:**
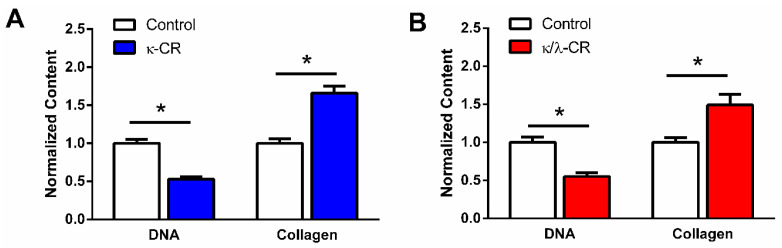
Effect of Hydrogel Confinement on DNA and Collagen Accumulation. DNA and collagen content were measured following 7 d in environment confined by either κ-CR (**A**) or κ/λ-CR hydrogels (**B**). Results were normalized to the Control (No CR) group. * indicates statistically significant difference between the CR group and Control (no-CR) group (*n* = 4, *p* < 0.05).

Having confirmed that hydrogel degradation supported sufficient CR release within the first 3 days of incubation to produce an MMC effect for a target protein (RNase A as a model protein), we evaluated the effects of the hydrogel-based crowding and confinement combination approach on collagen accumulation in tissue layers produced by the osteoblastic MG-63 cell line. Following 7 days of culture with the CR gel overlay (see Figure 1 for experimental set-up), cultures were analyzed via biochemical analyses following enzymatic digestion. Both hydrogel treatments (Figure 4A for κ-CR gel; Figure 4B for κ/λ-CR gel) resulted in significantly reduced DNA content but significantly increased collagen content compared to the cultures without any gel overlay. The DNA content provided an indirect measure of total cell number and was decreased by nearly half for each gel condition, indicating either cell death or decreased cell proliferation in the gel overlay culture. Note that visual observation of cells grown in washers only and in washers with a gel overlay did not indicate a negative effect of the experimental set-up on the 2D cell culture (Supplemental Figure S2). Furthermore, increase in DNA content was observed in the absence and presence of CR gel overlay, indicating cell proliferation over time (Supplemental Figure S3). The increase in collagen content relative to the control cultures was similar for both gels: 1.7-fold for the κ-CR gel treatment (Figure 4A) and 1.5-fold for the κ/λ-CR gel overlay (Figure 4B). To provide an estimation of collagen accumulation in the cultures on a per cell basis, the ratio of collagen content to DNA content was also determined. Compared to the paired unconfined control cultures, the collagen/DNA ratio was increased 3.2 ± 0.3-fold and 2.6 ± 0.2-fold with application of κ-CR or κ/λ-CR gels, respectively.

**Figure 5:**
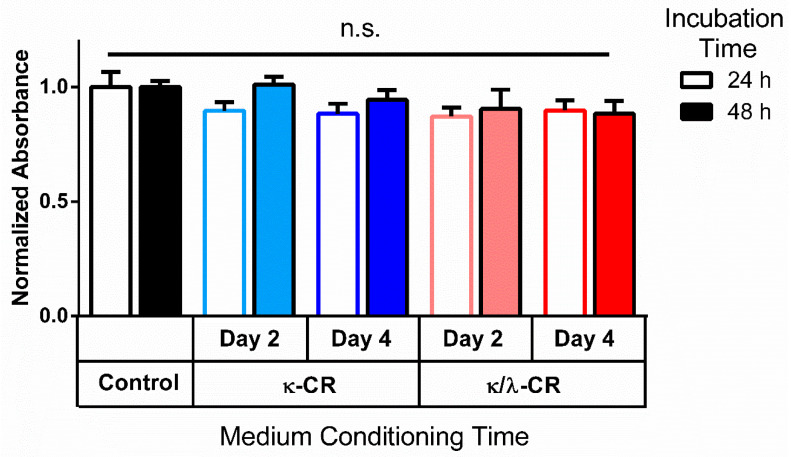
Metabolic Activity Following Incubation in Conditioned Medium. Metabolic activity was measured 24 h and 48 h after cell cultures were treated with medium conditioned by soaking κ-CR or κ/λ-CR hydrogels for two (D2) or four (D4) days in the incubator. Control group was unconditioned (no CR). Results were normalized to the control group for each time point. “n.s” denotes that no statistically significant difference was observed (*n* = 3).

Given the reduction in DNA content that occurred with gels placed above the MG-63 cell culture, a gel-conditioned medium experiment was conducted to assess whether the gel degradation products affected cell viability. Assessment of metabolic activity using a resazurin-based assay was performed following 24 and 48 h of treatment with gel-conditioned medium (Figure 5). Effects of gel-conditioned medium following incubation of the specific type of CR gel for 2 or 4 days at 37 °C were compared. There were no significant differences between absorbance values for the control culture and for any gel-conditioned medium group, indicating that the CR polymers released from the gels were not cytotoxic. Mean absorbance values for the treatment groups varied by less than 16% compared to the control cultures that received medium which had not been incubated with a gel.

### 2.4. Comparison of Soluble CR Isoforms on Collagen Deposition

**Figure 6:**
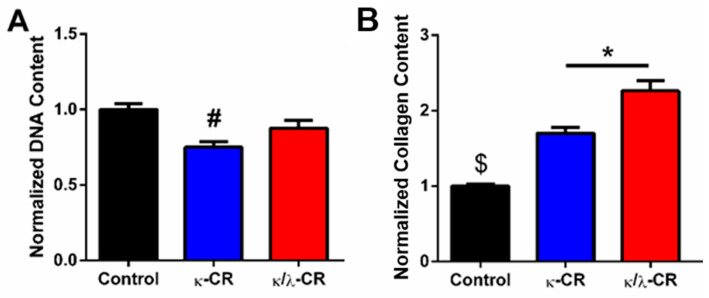
Effect of Extracellular Crowding by CR Isoforms. DNA content (**A**) and collagen content (**B**) were measured following 7 d of treatment with the indicated solubilized formulation (100 μg/mL). Results were normalized to the Control (No CR) group. # indicates statistically significant difference from the Control Group, $ indicates significant difference from all other groups, and * indicates significant difference between indicated groups (*n* = 4, *p* < 0.05).

To provide delineation of the effect of crowding (CR polymers released in the medium) versus confinement (CR gel overlay) for the CR hydrogel experiments, we conducted cellular experiments in which CR macromolecules were directly added to the culture medium to provide a baseline for effects of CR crowding alone with the MG-63 cells. Following 7 days of culture with the crowding reagent (100 µg/mL concentration), κ-CR significantly decreased DNA content by 25% compared to the untreated control culture (Figure 6A), while collagen content was significantly increased for each crowding treatment (Figure 6B). A 1.7- and 2.3-fold increase in collagen content relative to the control culture was observed in cultures treated with κ-CR or κ/λ-CR, respectively. The collagen/DNA ratio was increased 2.3 ± 0.2-fold with the κ-CR treatment and 2.5 ± 0.1-fold with the κ/λ-CR treatment compared to the control culture. This fold change is comparable to the level of change that occurred for the equivalent overlay cultures (see Figure 4). Overall, our results indicate that both carrageenan-based crowding and confinement approaches support increased collagen deposition by the MG-63 cultures.

### 2.5. Computaional Modeling of Glucose and Oxygen Transport

**Figure 7:**
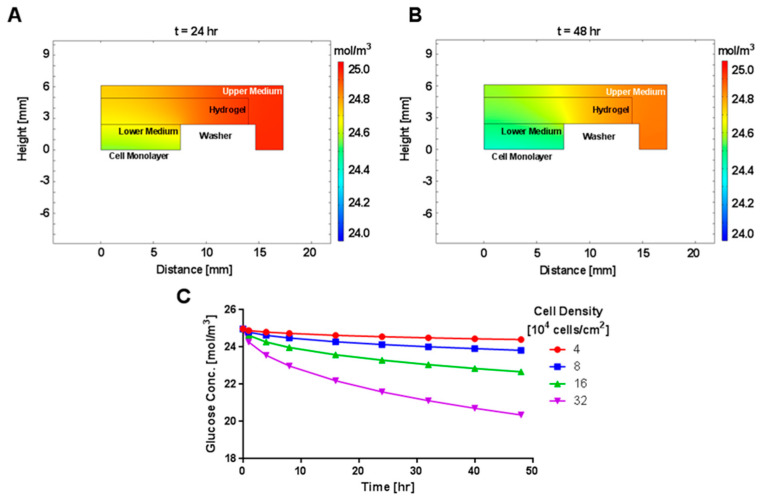
Computational Modeling of Glucose Concentration. Heat map of glucose concentration within model at 24 h (**A**) and 48 h (**B**) for cell density of 4 × 10^4^ cells/cm^2^. The washer was not included in the model, but its dimensions were used to define no-flux boundary conditions for the lower medium, hydrogel, and upper medium. (**C**) Calculated glucose concentration at varying cell densities as a function of time at the cell monolayer (height = 0) at the center of the well (radial position of 0).

The confinement culture system should sustain appropriate nutrient concentrations to support cell viability and procollagen secretion. We were concerned that the relatively large thicknesses of the three layers of the confined system (total transport distance of 6.1 mm) could have a negative impact on nutrient transport. Additionally, the nutrient consumption rates would be expected to change with time in culture, as the cell number increased due to proliferation. Given these considerations, models of glucose and oxygen transport within the confinement culture system were developed to quantify spatial variations in nutrient concentrations, and, in the case of glucose, to also describe time-dependent variations. Note that for all simulations we used cell densities 4- to 32-times higher than the initial seeding cell density to account for cell proliferation with time and to predict worst-case effects. Simulations with the time-dependent glucose transport model showed the reduction in glucose concentration over time through its consumption by the cell monolayer, which was represented as an outward flux at the lower boundary of the medium in contact with the cell layer. The culture medium formulation used for the cellular experiments was a high-glucose medium containing 4.5 g/L of glucose. All regions within the model started with a uniform glucose concentration of 25 mol/m^3^ (Supplemental Figure S4) because the hydrogels were soaked in culture medium before being used for cellular experiments. Concentration maps for the 4 × 10^4^ cell/cm^2^ cell density revealed that the glucose from the gel and medium directly above the cell layer was sufficient to meet the cellular demands in the first 24 h (Figure 7A), while the peripheral regions of the hydrogel and upper medium contributed to the glucose supply during the second 24 h (Figure 7B). As cell density was increased, the spatial variation of glucose concentration after 48 h of culture expanded to a larger region of the gel and the upper medium (Supplemental Figures S5). At 48 h, the minimum glucose concentration in contact with the cell layer was found to vary from 24.4 to 20.4 mol/m^3^ for cell densities ranging from 40,000 to 320,000 cells/cm^2^, respectively (Figure 7C). These changes represented a decrease of 2.3% to 18.5% relative to the initial concentration in the model. This minimum concentration was located at the center of the cell layer, and the glucose concentration in contact with the cell layer varied along the radial dimension from the center to the outer edge of the lower medium compartment by less than 0.9% for the three lowest densities and by less than 1.9% for the highest density (Figure 7). Overall, our simulation results indicated that at the cell densities used in this study (based upon cell growth from the initial 10,000 cells/cm^2^ seeding density) and with complete medium exchange every 2 days, the experimental set-up should not lead to significant glucose depletion.

**Figure 8:**
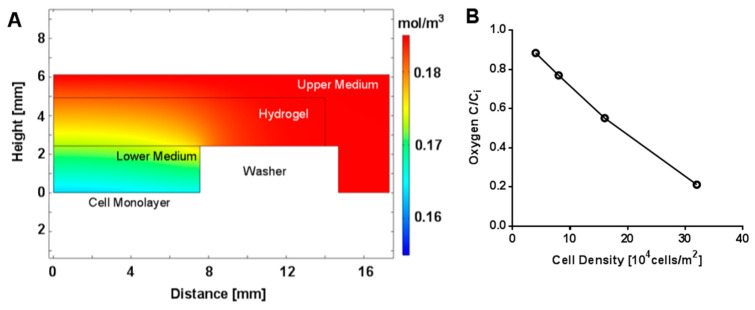
Computational Modeling of Oxygen Concentration. (**A**) Heat map of steady-state model of oxygen concentration at a cell density of 4 × 10^4^ cells/cm^2^. (**B**) Calculated oxygen concentration as a function of cell density at the cell monolayer (height = 0) at the center of the well (radial position of 0), where C_i_ is the initial concentration of oxygen at the source in contact with the upper medium boundary.

Effects of cell density on oxygen concentration were also evaluated in simulations of steady-state oxygen transport. With the oxygen supplied by the air in contact with the top boundary of the upper culture medium, spatial variation was confined to the regions of the model directly above the cell monolayer (Figure 8A and Supplemental Figure S6), where oxygen consumption was represented as an outward boundary flux. The ratio of oxygen concentration in the lower medium in contact with the cell layer to the source concentration was found to vary from 0.88 to 0.21 mol/m^3^ for cell densities ranging from 40,000 to 320,000 cells/cm^2^, respectively (Figure 8B), for the center of the well. Oxygen concentration in contact with the cell layer along the radial dimension increased steadily from the center of the well to the outer edge of the lower medium compartment and the amount of increase was influenced by cell density, with the two lower densities having a difference of less than 2.5% and the two higher densities having differences of 6.3% and 20.4%. Overall, our results again indicated that, at the cell densities used in this study, the cells should not be experiencing diminished oxygen levels in the experimental set-up.

**Figure 9:**
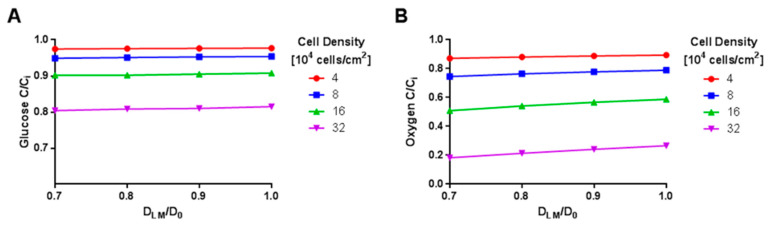
Effect of Hindered Diffusion on Solute Concentration. Calculated glucose (**A**) and oxygen (**B**) concentrations as a function of hindered diffusivity in lower medium (D_LM_) caused by crowding at t = 48 h. D_0_ refers to unhindered solute diffusion in aqueous medium. Concentrations were taken at the cell monolayer (height = 0) at the center of the well (r = 0).

The introduction of CR macromolecules into the culture medium through hydrogel degradation could affect diffusivity of nutrients within the culture medium. For each nutrient, simulations were conducted with the lower medium diffusivity reduced by 10% to 30% of the baseline diffusivity for the nutrient. Limited effects on glucose concentration in the medium at the cell layer were found at the 48 h time point (Figure 9A), with a 30% reduction in glucose diffusivity within the culture medium changing the concentration by less than 10% for the three lowest densities and by 20% for the highest density relative to the values occurring with the baseline diffusivity. Similar effects were seen when glucose diffusivity in the gel was reduced by up to 30% of the baseline diffusivity value (Supplemental Figure S7A).

The effect of reduced oxygen diffusivity within the lower medium on the steady-state oxygen concentration in the medium at the cell layer depended on cell density (Figure 9B). For the two lowest cell densities, a 30% reduction in oxygen diffusivity within the culture medium changed the concentration by less than 7% for the two lowest densities relative to the values occurring with the baseline diffusivity, but the two higher cell densities had changes of 50% and 82% relative to the baseline. Reduced oxygen diffusivity through the gel produced effects that were dependent upon cell density and of similar magnitude to those found with alternations in the lower medium diffusivity (Supplemental Figure S7B).

## 3. Discussion

This study demonstrated the potential for a combined confinement and crowding approach to enhance collagen deposition in a developing 2D tissue model using an osteoblast-like cell line. Our approach utilized a degradable CR hydrogel overlay to both offer a confinement barrier and be a source of MMC agents through degradation. Degradation of the CR hydrogels was characterized by both direct and indirect measurements, with similar levels of degradation found in both κ-CR and κ/λ-CR hydrogels. Hydrogel degradation led to release of soluble CR in the cell culture medium, which effectively slowed biomolecule transport in the medium as measured by FCS. We further showed that neither soluble nor hydrogel-released CR had a negative effect on cell viability, but both led to increase in collagen deposition by MG-63 cells. We also performed an in silico study to show that the confinement and crowding approach did not lead to depletion of oxygen and glucose available to the cells.

Given its demonstrated effectiveness as an MMC reagent and its prior use as a hydrogel, CR has several advantageous features as a biopolymer that could be used for the development of the combination crowding and confinement approach. CR hydrogels can be formed from the kappa and iota isoforms. Gelation is based upon physical crosslinking involving aggregation of double helices formed between adjacent polymer strands [27]. Gelation/aggregation is aided by the presence of a monovalent or divalent cation [28], although gelation can occur in the absence of ions. CR hydrogels are thermally reversible, and the gelation and melting temperatures depend upon the weight percentage of κ-CR and the ionic content of the solution [29]. The CR hydrogels evaluated in the study were prepared in DMEM or PBS, and each included potassium and sodium in the concentration ranges previously reported to support CR gelation. DMEM also included calcium ions that would have further supported hydrogel gelation and stability.

Incubation of the hydrogels at 37 °C in the confinement culture system was well below the predicted melting point, yet the CR hydrogels were found to undergo substantial degradation with incubations of less than 3 days. Hence, our results suggested that the thermal history of the CR hydrogels impacted the breakdown of the physical crosslinks. Additionally, the MG-63 cells potentially secreted factors that contributed to the breakdown of CR macromolecules, since we observed that gel degradation occurred more rapidly during cellular experiments than when incubated at 37 °C only in buffer or culture medium.

Degradation of both κ-CR and κ/λ-CR hydrogels was apparent after only 3 d of incubation at 37 °C, representing limited hydrogel stability. The degradation of CR hydrogels in the presence of PBS could be attributed to thermal degradation [30]. This degradation of CR hydrogels led to an increase in swelling ratio, indicative of a reduction in polymer crosslinking present within the hydrogel mesh network, which leads to higher swelling [31]. The loss of polymer content within the hydrogel also led to a decrease in measured hydrogel stiffness. Collectively, the analyses of swelling ratio and hydrogel stiffness indicated that the κ-CR hydrogel degraded more slowly than did κ/λ-CR hydrogel, suggesting enhanced network formation in the κ-CR hydrogels. Such an observation could be expected, as the λ-CR isoform is non-gelling. Therefore, at a given mass percentage, a hydrogel prepared from κ-CR would have more crosslinks formed than a hydrogel prepared from the κ/λ-CR mixture, leading to higher stability.

To understand the role of crowding, we studied how the MMC agents resulting from CR hydrogel degradation affected protein diffusivity in the medium above the cell culture. We used fluorescence correlation spectroscopy (FCS), which offers a unique opportunity to observe the diffusivity of molecules directly and in situ. Here, we used a model protein labeled with fluorescent dye to observe diffusion through an aqueous environment containing CR macromolecules created by degradation of the hydrogels. This model protein, RNase A (MW: 14.3 kDa), is smaller than a type I procollagen molecule (MW: 142 kDa) and would be expected to diffuse faster than would the procollagen [32]. However, a significant decrease in diffusivity for RNase A was still observed in hydrogel releasates for both κ-CR and κ/λ-CR. Therefore, a similar or even greater decrease in diffusivity would be expected for procollagen in these MMC environments [33]. The observed decrease in diffusivity corroborates our hydrogel degradation data, indicating that a sufficient mass of polymer chains was released from the bulk hydrogel over the course of 3 days to produce a MMC effect in solution.

The large dimensions of the gel used in the cellular experiments provided a total gel volume that was a significant source for CR macromolecule release. Based upon the mass release data obtained with smaller CR gels (50 µL volume), we estimate that the amount of CR mass released from the large gels (1850 µL volume) into the culture medium during cellular experiments would have produced a concentration of CR within the culture medium at least an order of magnitude higher than the upper range of concentrations used for CR in MMC experiments [16]. Future studies could also allow for direct measurement of procollagen diffusivity in either a crowded or confined volume, as well as within CR hydrogels. These experiments could elucidate the effectiveness of crowding and confinement to support enhanced procollagen processing and subsequent collagen fibril self-assembly.

Modifications to in vitro culture conditions, in which soluble neutral or negatively charged macromolecules are added to the culture medium, have been shown to enhance procollagen to collagen conversion and deposition in fibroblast cultures [34]. The soluble macromolecules occupy space in the dilute medium and increase the excluded volume, which may enhance protein–protein interactions and increase enzymatic activities [7]. Extracellular MMC using CR has been investigated in a variety of cell types and culture formats. Previous research has shown that CR is significantly more effective than Ficoll and dextrans, other common MMC agents, for increasing collagenous ECM deposition in WI-38 and WS-1 lung fibroblasts [15]. In the same study, CR was also shown to be effective on osteoblast and tenocyte culture’s fibrillar collagen production. In studies on dermal and corneal fibroblasts and human bone marrow mesenchymal stem cells, CR has been shown to increase the collagen deposition from 5- to 30-fold (depending on the cell type) [35,36,37].

Using a selectively permeable physical barrier to confine procollagens into a smaller volume of culture medium could be used as a complementary approach, along with the use of soluble MMC reagents, to support collagen fiber formation and enhanced ECM deposition during in vitro culture. A confinement barrier can be achieved in a variety of ways using encapsulation or entrapment within a gel or different forms of micropatterning [38,39]. This barrier can be applied using a gel overlay, which was used in at least one previous study [9]. In that study, an agarose gel was placed on a keratocyte monolayer and acted as a semi-permeable barrier to impede the diffusion of procollagens into the medium. This approach enhanced the processing of procollagen to collagen fibrils from 29% in standard cultures to 63–68% in cultures overlaid with agarose and fed with IGF-I and PDGF medium, and from 66% in standard cultures to 85% in cultures overlaid with agarose and fed with TGF-β medium.

Based on the above discussion, in this work we hypothesized that including confinement and crowding would combine the benefits of both and lead to similar or higher collagen deposition than MMC alone. As expected, both κ/λ-CR and κ-CR hydrogels were found to be effective in enhancing collagen deposition, with κ-CR gels being the more effective of the two. This could be due to the increased gel stability, resulting in different amounts of CR macromolecules in the medium above the cells. When used as soluble macromolecules of equal concentration, both types of CR were found to be equally effective in enhancing collagen deposition. Our results also indicated that collagen content was similar for both the combination approach and the MMC only approach. When normalized for cell number (DNA content), the ~3-fold change in collagen was similar to the results of others, where ~2–5-fold changes in collagen content were noted in the presence of MMCs compared to no MMCs [40]. While here we focused on collagen accumulation, future work could evaluate collagen distribution and types present, along with considering other components of deposited ECM, including fibronectin and non-collagenous bone-related proteins.

We also noted a decrease in DNA content for the combination approach at day 7, compared to cell culture with no crowding or confinement, indicating that fewer cells were present in the wells with the gel overlay. We confirmed that the soluble CR polymers released during gel degradation did not have a cytotoxic effect on the cells, which was expected based on their successful use as MMC agents. We further confirmed that cells were viable and increasing in number for both conditions during the 7-day culture period, but that MG-63 cells had slower proliferation rates in the gel overlay cultures. Cell imaging also showed that cells had similar morphology for both conditions, with no visible signs of cells dying. It is of note, however, that while MG-63 cells deposit collagen, they are also an osteosarcoma cell line and are not growth inhibited; hence, reaching confluency over the 7-day growth period was not expected to impact their cell viability. The computational modeling simulations of nutrient transport within the confined culture system in parallel with nutrient consumption by the cell monolayer demonstrated that oxygen and glucose concentrations at all locations within the medium remained at sufficient levels to support cell viability, even at the highest cell density evaluated. Simulations were performed across a range of cell densities to account for increased nutrient demand caused by MG-63 cell proliferation throughout the culture period. Together, these multiple analyses indicate that cell death was unlikely to have significantly contributed to the reduction in cell number.

Two plausible explanations for the lower cell numbers would be a reduction in the proliferation rate or a loss of cells from the culture well surface caused by a disruption in cell adhesion, with either effect resulting from soluble CR macromolecules within the culture medium. It has been previously reported that negatively charged macromolecules can interfere with cell adhesion and cause cell detachment through electrostatic interactions [7,8]. Given the very high concentration of CR that we estimated to be present in the lower medium based upon the CR mass release data (see earlier paragraph for more detailed description), clumping of the CR macromolecules was likely and this could have led to settling of CR directly onto the cell monolayer. A direct interaction of CR molecules with the plasma membrane would provide a pathway for disruption of various cellular processes, including adhesion or proliferation.

While more work is needed to clearly show whether using confinement and crowding together is superior to an MMC-only approach, the combination approach described here did significantly increase collagen production, especially on a per cell basis, and has some advantages. First, a degradable CR gel is a semi-continuous source of MMCs, where new MMCs are released in the medium in a sustained manner. This could ameliorate possible issues with macromolecules settling out of solution and depositing on the cell monolayer or the need to continuously supplement the culture with macromolecules. Presenting both confinement and crowding is also physiologically relevant and similar to the native cell environment. Although our long-term research focus would be to generate a periosteum using a human mesenchymal stem cell source, the use of the MG-63 cell line was a reasonable choice for a proof-of-concept evaluation of the combination approach, and avoided any of the complicating effects of MMCs on osteoblast differentiation of human mesenchymal stem cells that have been noted by others [41].

## 4. Conclusions

In conclusion, here we developed a combined confinement and crowding approach using a degradable CR hydrogel. The hydrogel was overlayed on a 2D culture of MG-63 cells through a spacer and provided both medium confinement and a source for sustained CR polymer release. Our combined approach resulted in ~3-fold change in normalized collagen content and performed similarly to a crowding only approach. While the combined approach seemed to slow cell proliferation, it did not affect cell viability, morphology, or access to oxygen and nutrients. The combined approach is promising for various tissue engineering applications, where generating thin cell-secreted tissues that are compositionally and structurally similar to native tissues is desired.

## 5. Materials and Methods

### 5.1. Materials

The culture medium, other supplements for cell culture including CR macromolecules (catalog # 22048 for κ-CR and # C1013 for the κ/λ-CR mixture; Figure 1A), and chemical reagents for assays were from Sigma Aldrich Co. (St. Louis, MO, USA), except where noted otherwise. The MG-63 osteosarcoma cell line was from ATCC^®^ (Catalog # CRL-1427^™^; Manassas, VA, USA). Bovine serum was from Atlas Biological, Inc. (EquaFetal, Fort Collins, CO, USA). Ultra-high molecular weight polyethylene (UHMWPE) washers (for 5/8” Screw Size with 0.64” ID, 1.062” OD, and 0.28” thickness) were from McMaster-Carr (Elmhurst, IL, USA). Sylgard 184 silicone elastomer was from Dow Corning Corporation (Midland, MI, USA). Fluorescent dye removal columns, Atto 655 NHS-ester, and the Quant-iT^TM^ PicoGreen^®^ dsDNA Reagent kit were from Thermo Fisher Scientific (Waltham, MA, USA). CoverWell perfusion chamber gaskets and silicon spacers were from Grace Bio-Labs (Bend, OR, USA).

### 5.2. CR Hydrogel Formation

A 5% *w*/*v* κ-CR or κ/λ-CR solution was prepared by combining CR powder with high-glucose Dulbecco’s Modified Eagle Medium (HG-DMEM) and stirring at 100 °C until homogeneous. Heating CR solutions to temperatures above 80 °C allowed for a CR solution to form and is a well-established method for fabricating CR hydrogels (Supplemental Figure S8) [42,43]. The CR solution was poured into wells of a 6-well culture plate to form gel layers (~2–3 mL/well), and then a circular punch (2.8 cm diameter) was used to create CR gels (thickness ~2–3 mm) for cellular experiments. For sterilization, gels were soaked in ethyl alcohol at 4 °C overnight, followed by three rinses in 1× Dulbecco’s Phosphate Buffered Saline (DPBS) and ultraviolet exposure for 6 h within the biosafety cabinet. This sterilization was shown to not impact hydrogel mechanical properties (Supplemental Figure S1). Hydrogels were subsequently stored at 4 °C in HG-DMEM supplemented with 5% *v*/*v* bovine serum and 1% *v*/*v* penicillin/streptomycin for 2–4 days prior to use in cellular experiments.

### 5.3. Characterization of Hydrogel Degradation

κ-CR and κ/λ-CR hydrogels (5% *w*/*v*) were formed as described above. Initial mass, *M*_0_, of each gel was measured with a Mettler Toledo XS104 Balance (Columbus, OH, USA). Hydrogels were then incubated in 24-well plates with PBS for 1–3 days at 37 °C. Hydrogels were removed from the PBS, gently patted with a Kimwipe to remove excess liquid, and swollen mass, *M_s_*, was measured. Hydrogels were then dried for 24 h at 60 °C to obtain the dry mass, *M_D_*. Swelling ratio *Q_M_*, was calculated as *Ms/M_D_*. Hydrogel mechanical properties were measured using an AR 2000ex Rheometer (TA Instruments, New Castle, DE, USA) with a 20 mm parallel plate geometry. Hydrogels were prepared, cut into 1 × 20 mm slabs, and incubated in PBS at 37 °C for 1, 2, or 3 days. Storage modulus, *G*′, and loss modulus, *G*″, were measured as a function of angular frequency from 1–10 rad/s.

To directly measure the degradation of CR hydrogels, a release study was performed. Hydrogels were prepared (50 µL volume) and incubated in 4 mL of PBS at 37 °C on a rocking platform. At specified time points, 500 µL of release buffer was collected and replaced with fresh PBS. Samples were stored at 4 °C for up to one week prior to analysis. CR released from hydrogels was quantified by measuring absorbance at 500 nm with a SpectraMax i3 plate reader (Molecular Devices, San Jose, CA, USA). Standard curves were developed using solubilized 0–1% *w*/*v* κ-CR or κ/λ-CR in PBS, while a mass balance was utilized to calculate the total mass of released CR at each time point as follows:

(1) $$\frac{M_{i}}{M_{\infty }}=C_{i}V+\sum C_{i-1}V_{s}$$

where *M_i_* is the mass released at time *i*, *M_ꚙ_* is the initial concentration of CR within the hydrogel (5% *w*/*v*), *C_i_* is the concentration of CR released at time *i*, *V* is the total volume of the release solution (4 mL), and *V_s_* is the releasate sample volume (0.5 mL).

### 5.4. Florescence Correlation Spectroscopy

Fluorescence correlation spectroscopy (FCS) was performed to demonstrate the crowding effect from hydrogel degradation. First, ribonuclease A (RNase A) was fluorescently labeled with Atto 655-NHS ester, according to the manufacturer’s protocol with 64% labeling efficiency. Briefly, fluorescent dye and RNase A were dissolved in PBS and left to react with mixing for 2 h, while protected from light. Unbound dye was removed using dye removal columns with > 95% removal efficiency.

Next, κ-CR and κ/λ-CR hydrogels (5% *w*/*v*) were incubated in 3 mL of PBS at 37 °C for 1, 2, or 3 days. Releasate samples were removed and stored at 4 °C until FCS analysis. FCS samples were prepared by gently mixing 100 µL of hydrogel releasate or PBS with Atto 655-labeled RNase A for a final protein concentration of 1 µg/mL. Sample solutions (40 µL) were loaded into perfusion chamber gaskets adhered to a #1.5 coverslip and capped to avoid evaporation. FCS measurements were performed using Microtime200 software (PicoQuant, Berlin, Germany). Atto 655 (0.2 nM) in PBS was used to calibrate the FCS instrument confocal volume. A 640 nm ps pulsed laser was used at an optical power of ~11.4 µW for at least six measurements of 180 s for each sample. An autocorrelation function *G*(*τ*) was obtained for each measurement:

(2) $$G\left(\tau \right)=\frac{1}{N}\frac{1}{\left[1+\left(\frac{\tau }{\tau_{D}}\right)\right]}\frac{1}{{\left[1+p\left(\frac{\tau }{\tau_{D}}\right)\right]}^{0.5}}$$

where *n* is the number of fluorescent particles, *p* = *r_o_/z_o_* is an instrumental constant, *r_o_* is the radius and *z_o_* is the axial length of the focused laser beam spot, and *τ_d_* is the solute diffusion time. A one component autocorrelation function fit was used for all samples. Additionally, a triplet model was used to account for possible excitation of molecular triplet states at higher laser intensities. Autocorrelation functions for labeled RNase A were normalized as follows:

(3) $$Normalized\;G\left(\tau \right)=\frac{G(\tau_{D})}{G(\tau_{0})}$$

where *G*(*τ_D_*) is the value of the Equation (3) at each time point and *G*(*τ*_0_) is the value of Equation (3) at the initial time point. The effective tracer diffusion coefficient for each protein in solution was calculated from *τ_D_* as follows [44]:

(4) $$D_{FCS}=\frac{{\left(r_{0}\right)}^{2}}{4\tau_{D}}$$

### 5.5. Cellular Experiments with CR Hydrogels

The MG-63 osteosarcoma cell line was expanded up to passage 9 in growth medium that contained HG-DMEM with 5% *v*/*v* bovine serum and 1% *v*/*v* penicillin/streptomycin, with culture at 5% CO_2_ and 37 °C. For all experiments, cells were harvested by a 5 min exposure to Trypsin/EDTA prior to seeding. Experiments were performed in 6-well plates containing UHMWPE washers glued to the bottom of each well using Sylgard 184 with 4 h curing at 37 °C (Figure 1B). MG-63 cells (10,000 cells/cm^2^) were seeded within the inner diameter of the washer in 0.5 mL of growth medium and cultured for 3 days at 37 °C and 5% CO_2_. The medium was then replaced with growth medium supplemented with 0.25 mM ascorbic-acid-2-phosphate (AA2P), a hydrogel was placed upon the washer for the treatment groups, and additional medium (2.5 mL) was added to the well to cover the hydrogel. Medium and gels were replaced once every 2 days using a total feed volume of 3 mL per well for all groups.

### 5.6. Biochemical Analyses for Collagen and DNA Content

For the cellular confinement and soluble MMC experiments, cultures were rinsed with DPBS and incubated in digest solution (pH 7.0) containing 0.25 mg/mL papain, 80 mM sodium phosphate, 6 mM EDTA, and 5 mM cysteine hydrochloride at room temperature on a shaker plate for 10 min to promote detachment. Next, the contents from each well were transferred into a microcentrifuge tube and placed at 60 °C overnight. A modified colorimetric chloramine T-based assay was used to measure hydroxyproline content in the digested samples [45]. Briefly, equal volumes of digested samples and 4N NaOH were combined and then incubated for 25 min at 120 °C and 15 psi. After cooling to ambient temperature, samples were neutralized by the addition of 4N HCl. Next, a chloramine T-based solution was added to the samples and incubated for 20 min, followed by addition of an Erlich (4-Dimethylamino benzaldehyde) solution. After incubation at 65 °C for 20 min, the absorbance of samples was read at 550 nm with a SpectraMax i3 plate reader and compared to a standard curve prepared from trans-4-hydroxy-L-proline. Hydroxyproline content was used as an indirect measurement of collagen content. DNA content in the digested samples was measured using the Quant-iT^TM^ PicoGreen^®^ dsDNA Reagent kit as per the manufacturer’s protocol. After incubation in the reagent for 5 min, fluorescence intensity was measured at 480 nm and 520 nm for excitation and emission wavelengths, respectively, with a SpectraMax i3 plate reader. DNA content was analyzed as an indirect measurement of total cell number.

### 5.7. Resazurin Assay for CR Gel Toxicity

Resazurin assay was used to measure CR gel toxicity [46]. κ-CR and κ/λ-CR hydrogels were prepared as described previously and placed into 6-well plates with 3 mL of growth medium per well. Gels were incubated in the medium for 2 or 4 days at 37 °C, with the CR-gel conditioned medium collected following incubation and stored at −80 °C until use. A control medium-only group (no gel) was also incubated for 2 days and stored at −80 °C until use. For cytotoxicity testing, MG-63 cells were seeded in a 96-well plate (10,000 cells/cm^2^) in growth medium; then, after 24 h, growth medium was replaced with gel-conditioned medium and cultured for another 24 or 48 h. Prior to use, the conditioned medium samples were thawed in a 37 °C water bath and then supplemented with 2.5% bovine serum to provide sufficient nutrition for the MG-63 cultures. At 24 or 48 h after addition of the gel-conditioned medium, the conditioned medium was replaced with 100 µL growth medium and 10 µL of 0.15 mg/mL resazurin reagent prepared in HG-DMEM. Following a 2 h incubation at 37 °C and 5% CO_2_, an 80 µL sample was taken from each well and transferred into a new 96-well plate, and fluorescence intensity was measured at an excitation/emission wavelength of 560/590 nm with a SpectraMax i3 plate reader.

### 5.8. Soluble Crowding Experiments

Stock solutions of CR macromolecules were prepared in DPBS (5 mg/mL for κ-CR; 2.5 mg/mL for κ/λ-CR) and autoclaved for sterilization. MG-63 cells (10,000 cells/cm^2^) were cultured in 12-well plates in “growth medium” for 3 days, as described previously in Section 5.5. The medium was then replaced with growth medium supplemented with 0.25 mM AA2P. For each treatment group, an appropriate volume of stock solution of the specified CR macromolecule was also added to the culture medium to yield a CR concentration of 100 μg/mL. Cultures were continued for 7 days, with medium replaced on days 3 and 5.

### 5.9. Computational Modeling of Glucose and Oxygen Transport

A 2D axisymmetric computational model of the hydrogel confinement culture system was developed (COMSOL software, V5.6, COMSOL Inc., Stockholm, Sweden) to simulate Fickian diffusion of a dilute species. Geometric dimensions of the model are given in Supplemental Table S1. Glucose transport was evaluated using a transient model (Equation (5)):

(5) $$\frac{\partial c_{A}}{\partial t}=D_{AB}\left(\frac{1}{r}\frac{\partial }{\partial r}\left(r\frac{\partial c_{A}}{\partial r}\right)+\frac{\partial^{2}c_{A}}{\partial z^{2}}\right)$$

Glucose transport simulations were conducted for a 48 h period using a time step of 300 s. The initial value for the concentration of glucose in the culture medium was 4.5 g/L to match the medium formulation in the cellular experiments, and this was applied to all regions of the culture medium and the hydrogel. Diffusivity of glucose in the culture medium was assumed equal to the diffusivity of glucose in water at 37 °C, given as 6.14 × 10^−10^ m^2^/s by Zhou et al. [47]. Considering the low weight percentage of CR molecules used to formulate the hydrogel layer in the cellular experiments, glucose diffusivity in the hydrogel layer was also assumed to be equal to its diffusivity in water at 37 °C. Simulations of oxygen transport used a steady-state transport model (Equation (6)) due to the infinite source of oxygen from air within the incubator.

(6) $$0=\frac{1}{r}\frac{\partial }{\partial r}\left(r\frac{\partial c_{A}}{\partial r}\right)+\frac{\partial^{2}c_{A}}{\partial z^{2}}$$

where *r* is the radius, *c_A_* is the concentration of oxygen, and *z* is the distance in the z direction.

The oxygen source was represented as a constant concentration boundary condition (0.185 mol/m^3^) at the upper surface of the culture medium assuming a 21% partial pressure gradient of oxygen in dry air and an 80% relative humidity in the incubator, based upon the values given by Brown et al. [48]. Diffusivity of oxygen in the culture medium and in the hydrogel layer were both assumed equal to the diffusivity of oxygen in water at 37 °C, given as 2.7 × 10^−9^ m^2^/s [48]. For both transport models, the edges of the culture medium and hydrogel in contact with the washer and the well dish were modeled as no-flux boundaries. Nutrient consumption by the cell layer at the lower surface of the model was represented as a flux boundary based upon Michaelis–Menten kinetics (Equation (7)):

(7) $$N_{i_{z}}=-\sigma_{Cell}V_{M}\frac{c_{i}}{K_{M}+c_{i}}$$

where $V_{M}$ is the maximum reaction rate, $K_{M}$ is the Michaelis constant representing the half-maximum concentration for the reaction, and $\sigma_{Cell}$ is the cell density at the bottom of the well. The values used for glucose were 8.6 × 10^−17^ for $V_{M}$ [49] and 0.35 mol/m^3^ for $K_{M}$ [50], while the values used for oxygen were 2.9 × 10^−17^ for $V_{M}$ [51] and 0.01105 for $K_{M}$ [52]. Values of 40, 80, 160, and 320 × 10^7^ cells/m^2^ were evaluated for $\sigma_{Cell}$.

### 5.10. Statistical Analyses

Results are presented as the mean ± standard error of the mean. Each experiment was repeated at least 3 times, with a minimum of 3 samples per group. A *t*-test was used for comparisons involving a single treatment group and a one-way analysis of variance (ANOVA) was used to compare means of groups for experiments involving multiple treatments. When ANOVA showed a significant difference, a Tukey post-hoc test was used for pairwise comparisons. Statistical analyses were performed using a 95% confidence level (GraphPad Prism 10.0.2 software, Boston, MA, USA).

## Data Availability

The data are contained within the article or Supplementary Material.

## Supplementary Materials

The following supporting information can be downloaded at: https://www.mdpi.com/article/10.3390/gels9090705/s1, Figure S1: UV Sterilization Did Not Affect CR Hydrogel Stiffness; Figure S2: Brightfield Cell Proliferation Images in the Presence of CR Hydrogels; Figure S3: Quantification of DNA Content to Demonstrate Cell Proliferation in Washer and Gel System. Figure S4: Heat Map of Initial Glucose Concentration; Figure S5: Heat Map of Glucose Concentration at t = 48 h for Varying Cell Densities; Figure S6: Heat Map of Oxygen Concentration at t = 48 h for Varying Cell Densities; Figure S7: Effect of Gel Diffusivity on Solute Concentration; Table S1: Geometric Dimensions of Computational Model; Figure S8: Dependence of CR Stiffness on Temperature.
