# Supplementary material for: Carrageenan-Based Crowding and Confinement Combination Approach to Increase Collagen Deposition for In Vitro Tissue Development
Source: Gels. 2023 Sep 1;9(9):705. doi: 10.3390/gels9090705 (PMC10529090; doi:10.3390/gels9090705)
Supplement: Supplementary file 1 [file gels-09-00705-s001.zip › gels-2557160-supplementary.pdf]

Supplemental Information

Carrageenan-based Crowding and Confinement Combination Approach to Increase Collagen Deposition for *In Vitro* Tissue Development

Joseph Krebs [1], Samuel Stealey [1], Alyssa Brown [1], Austin Krohn [1], Silviya P. Zustiak [1,2], and Natasha Case [1,*]

[1] Department of Biomedical Engineering, Saint Louis University, Saint Louis, MO, USA
[2] Department of Physiology and Pharmacology, School of Medicine, Saint Louis University, Saint Louis, MO, USA
* Correspondence: Email: natasha.case@slu.edu; Tel: 314-977-8646

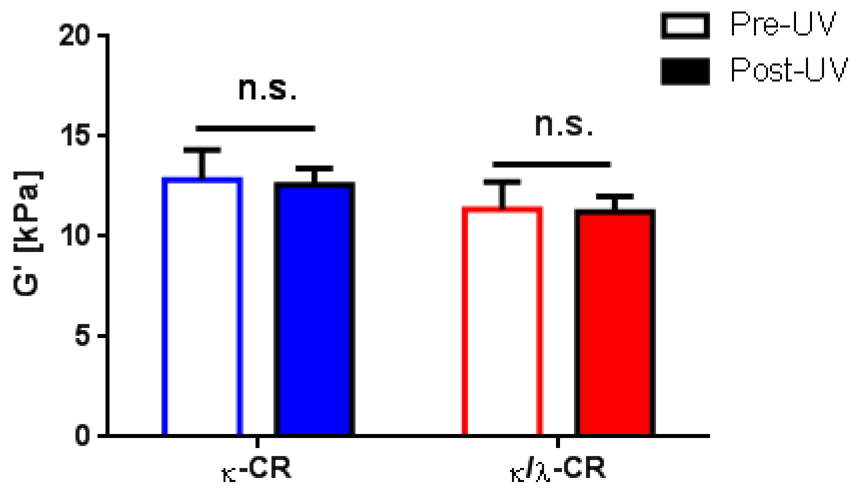

Figure S1. UV Sterilization Did Not Affect CR Hydrogel Stiffness. CR hydrogels were tested for stiffness (as quantified by storage modulus, *G′*, prior to- and following UV sterilization procedure.

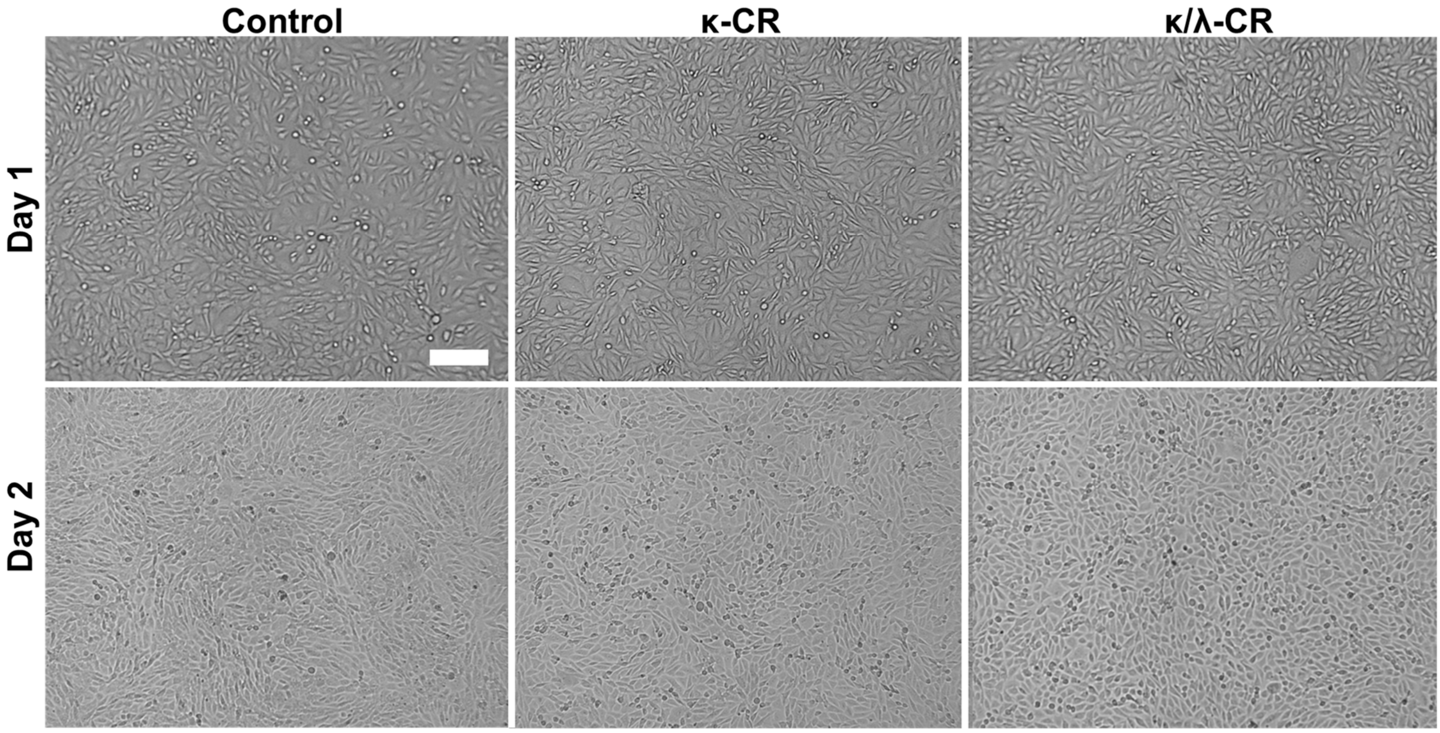

Figure S2. Brightfield Images of MG-63 Cells Proliferating in the Absence (Control) or Presence of κ-CR or κ/λ-CR Hydrogel. Scale bar represents 50 μm.

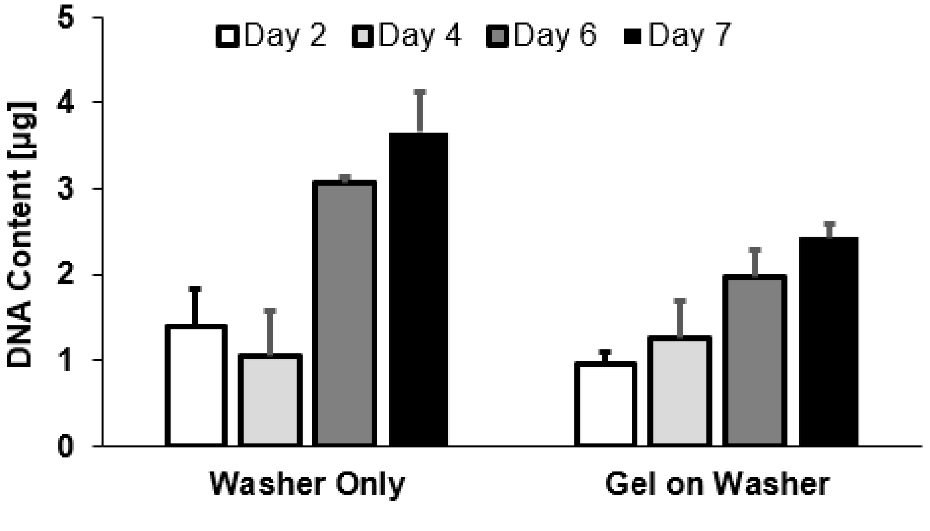

Figure S3. Time Course Evolution of Normalized DNA Content in the Absence (Washer Only) and Presence (Gel on Washer) of a 5% κ-CR Gel.

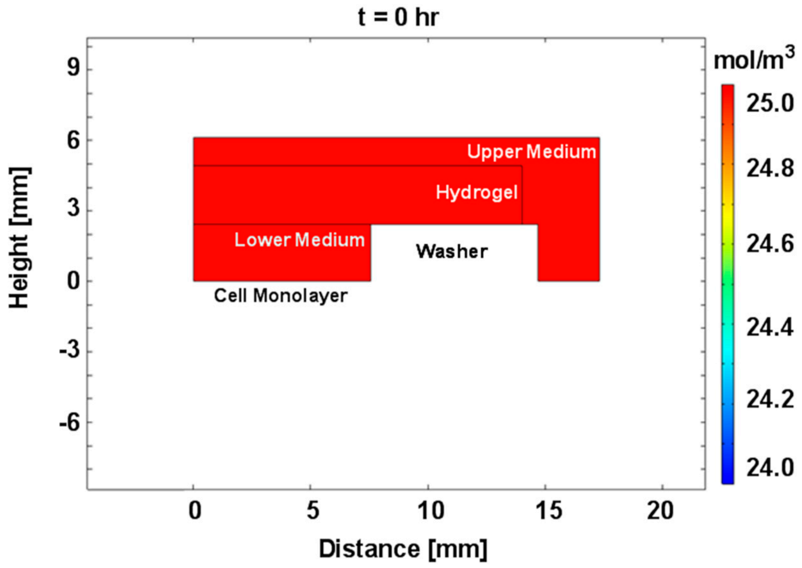

Figure S4. Heat Map of Initial Glucose Concentrations at t = 0 h.

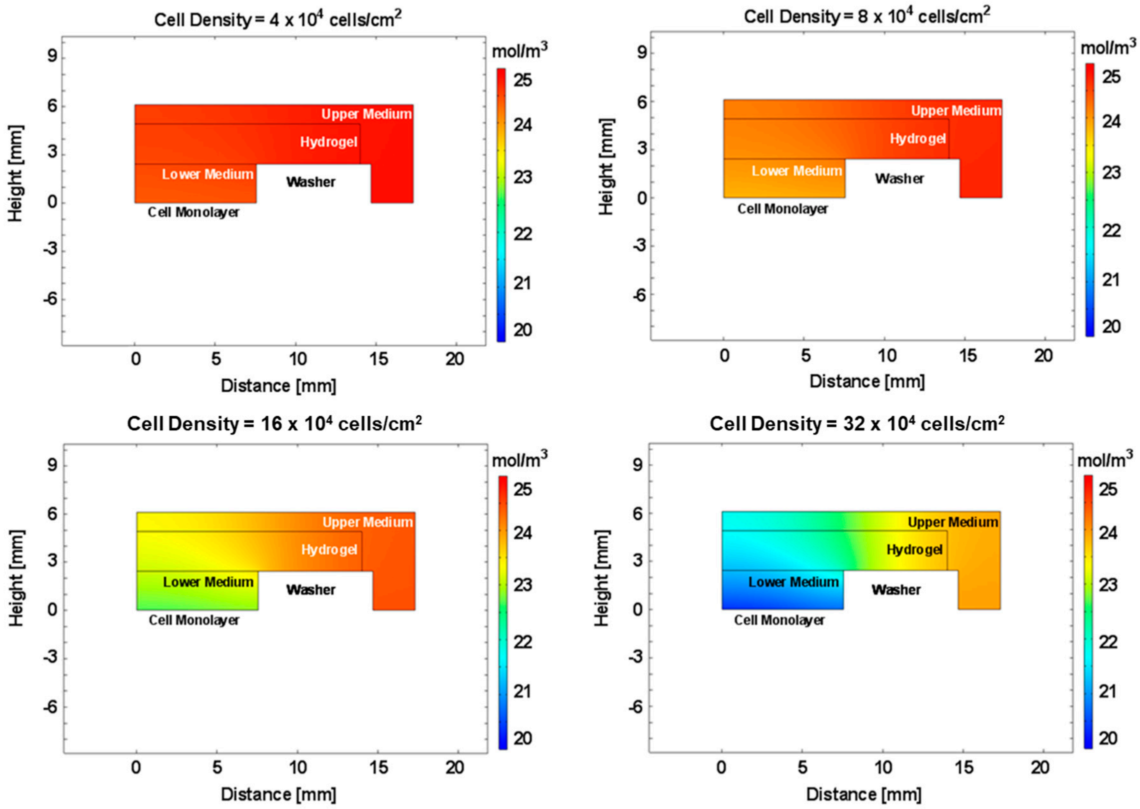

Figure S5. Heat Map of Glucose Concentrations at t = 48 h for Varying Cell Densities.

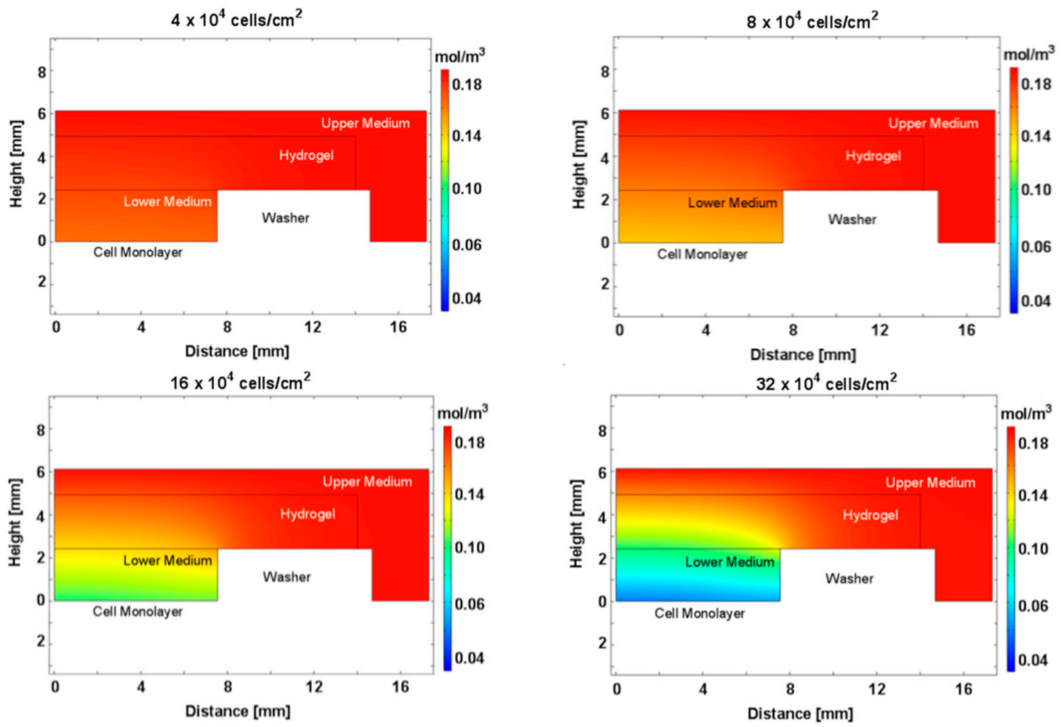

Figure S6. Heat Map of Oxygen Concentrations for Varying Cell Densities.

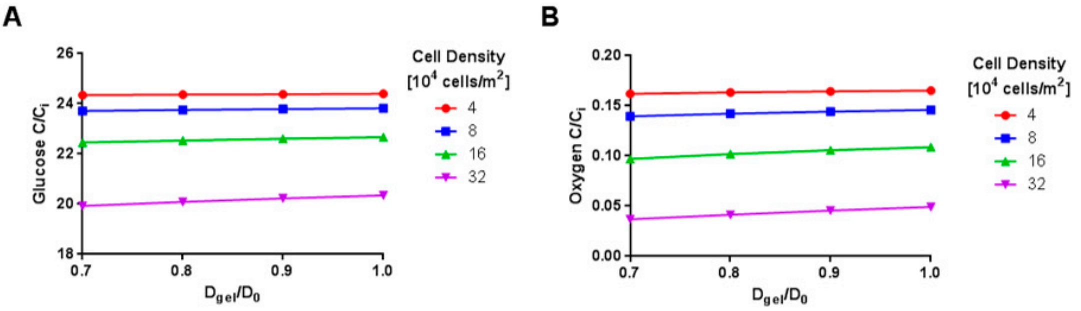

Figure S7. Effect of Gel Diffusivity on Solute Concentration. Calculated glucose (**A**) and oxygen (**B**) concentrations as a function of diffusivity through the hydrogel caused by changing effective polymer concentrations (D_{gel}). Here, D_0 refers to unhindered solute diffusion in aqueous media and C_i is the initial concentration of glucose or oxygen at t = 0 hr. Concentrations were taken at the cell monolayer (height = 0) at the center of the well (r = 0).

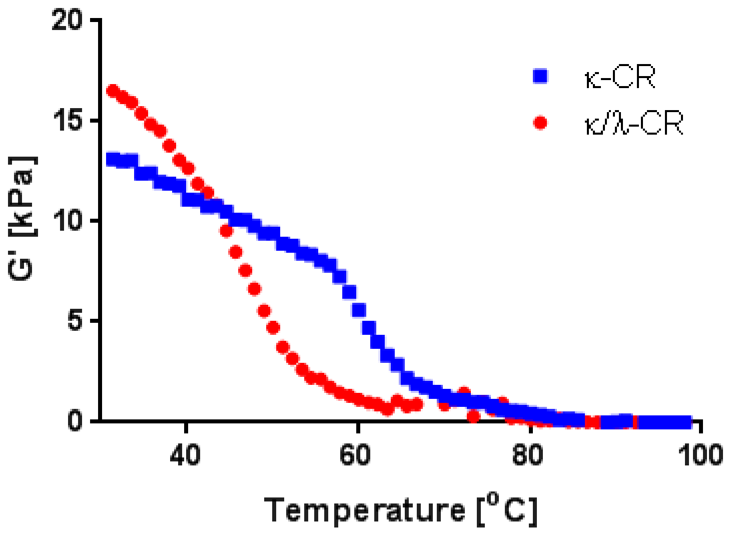

Figure S8. Dependence of CR Stiffness on Temperature. CR solutions were brought to 100 °C and cooled at a rate of 10 °C per minute while storage modulus, *G′*, was constantly measured using a parallel plate geometry rheometer with a constant strain of 0.5% and angular frequency of 1 rad/s.

Table S1. Geometric dimensions of computational model of hydrogel confinement culture system.

Parameter	Description	Value	Units
r_{UM}	Upper medium radius	0.0173	m
r_H	Hydrogel radius	0.014	m
r_{LM}	Lower medium radius	0.0075	m
t_W	Distance between radial boundary of lower medium and inner radial boundary of lower region of upper medium*	0.00713	m
h_{UM}	Upper medium height above hydrogel	0.0012	m
h_H	Hydrogel height	0.0025	m
h_{LM}	Lower medium height	0.0024	m

*Note: dimension is equal to radial thickness of washer used in cellular experiments
